# Patient-derived xenografts: a relevant preclinical model for drug development

**DOI:** 10.1186/s13046-016-0462-4

**Published:** 2016-12-05

**Authors:** Luca Pompili, Manuela Porru, Carla Caruso, Annamaria Biroccio, Carlo Leonetti

**Affiliations:** 1 UOSD SAFU, Department of Research, Diagnosis and Innovative Technologies, Traslational Research Area, Regina Elena National Cancer Institute, Via Elio Chianesi 53, Rome, 00144 Italy; 2 University of Tuscia, Viterbo, Italy; 3 Oncogenomic and Epigenetic Unit, Regina Elena National Cancer Institute, Rome, Italy

**Keywords:** Patient-derived-xenografts, Immunosuppressed mice, Subcutaneous, Orthotopic, Tumor heterogeneity, Predictive value

## Abstract

Identifying appropriate preclinical cancer models remains a major challenge in increasing the efficiency of drug development. A potential strategy to improve patient outcomes could be selecting the ‘right’ treatment in preclinical studies performed in patient-derived xenografts (PDXs) obtained by direct implants of surgically resected tumours in mice. These models maintain morphological similarities and recapitulate molecular profiling of the original tumours, thus representing a useful tool in evaluating anticancer drug response. In this review, we will present the state-of-art use of PDXs as a reliable strategy to predict clinical findings. The main advantages and limitations will also be discussed.

## Background

Ever since the first studies reported on the use of *in vivo* murine leukemia models for drug efficacy in the 1950s [1], vast efforts have been devoted to the development of animal models in cancer to predict the response of chemotherapeutics in humans. The introduction of a variety of immuno-deficient mice enabled us to engraft tumour cell lines by ectopic or orthotopic injection. While this approach allows many models to be established with relative ease, these xenografts bear little resemblance with the original tumours, in terms of molecular complexity and tumour heterogeneity. It is for these reasons, that the use of these models in evaluating novel agents is limited and can account for the strong discrepancy between preclinical efficacy and clinical response for cancer disease [2].

In recent years, patient-derived xenografts (PDXs), where tumour fragments from patients are directly implanted in immunodeficient mice and then passed *in vivo* directly from mouse to mouse, have emerged as important tools for translational research. PDXs maintain the cellular and histological structure of the original tumour and include critical stromal elements, which provide sustenance under periods of extensive growth [3]. Moreover, cytogenetic analysis of tumours from PDXs revealed strong preservation of the overall genomic and gene expression profile of the corresponding patient tumours [4, 5]. Interestingly, the response/resistance of PDXs to standard chemotherapeutics or targeted compounds closely correlated with clinical data in patients from which PDXs had been derived [6, 7]. All these characteristics highlight the use of PDXs as more predictive experimental models for evaluating therapeutic responses.

## Generation of PDXs

PDXs are developed by implanting fresh human tumour fragments in immunosuppressed mice. Usually, the time required for the tumour to take is between 2–4 months, although failure of engraftment should not be ascertained until at least 6 months and beyond [3].

**Fig. 1:**
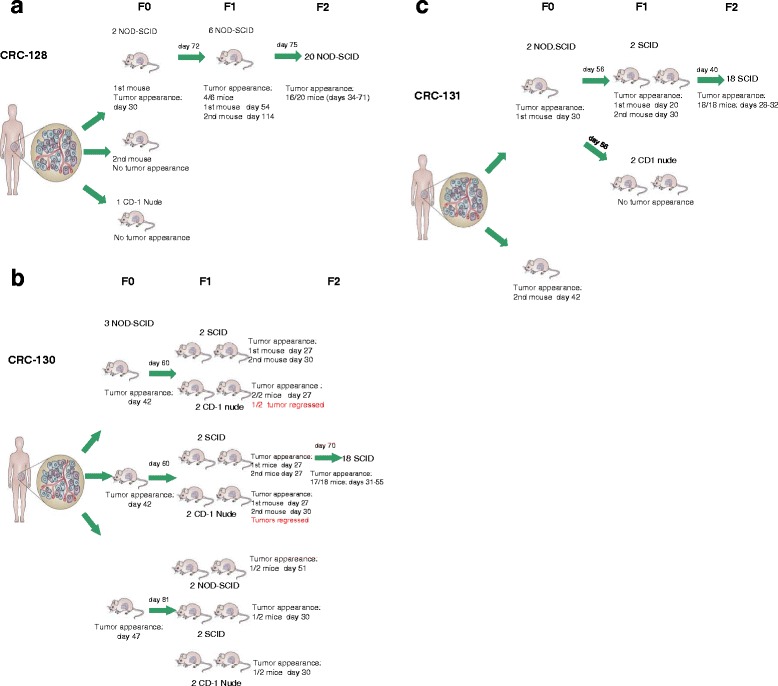
Figure show the take rate obtained by the implantation of tumor fragments from patients with colorectal cancer in different strains of immunodeficient mice. In particular, fragments from primary tumors of patient 128 (**a**) and of patient 130 (**b**) or from a metastatic limph node of patient 131 (**c**) were placed in medium supplemented with antibiotics, diced into 15–20 mm^3^ pieces, coated in Matrigel and implanted by a small incision and subcutaneous pocket made in one side of the lower back into different mice (*F0*). After tumor mass formation, at the indicated day, tumors were passaged (*F1*) and expanded in large cohorts for drug sensitivity experiments (*F2*). All animal procedures were approved by the ethics committee of the Regina Elena National Cancer Institute (CE/534/12) and were in compliance with the national and international directives (D.L. March 4, 2014, no. 26; directive 2010/63/EU of the European Parliament and of the council; Guide for the Care and Use of Laboratory Animals, United States National Research Council, 2011)

The mice strains used for tumour initiation and propagation are: i) nude mice, which lack a thymus and are unable to produce T cells; ii) NOD-SCID and SCID-beige mice, which lack functional T, B and NK cells; iii) NOD-SCID IL2RGamma null (NOD-SCID Gamma, NSG), in which the NK cell activity is completely absent. Due to different immunological impairments, it is assumed that the more permissive mouse strains such as NOD-SCID, SCID or NSG, can strongly increase the efficiency of xenotransplantation, as compared to nude mice. Indeed, a very low tumour rate take (10–25%) was reported after implanting tumour fragments of different histotypes in nude mice [8–10]. The use of NOD-SCID resulted in an increased engraftment rate (25–40%) for non-small cell lung cancer, breast cancer and melanoma [11–15] and a very high tumour take-rate (from 50 to 80%) has been observed for ovarian cancer, head and neck tumours, metastatic colon and bladder cancer [6, 16–18]. In our experience, to establish colon cancer PDXs, we observed that implanting fragments in nude (nu/nu) mice did not produce tumour growth neither at F0 (Fig. 1a) nor at F1 passages (Fig. 1b and c). Moreover, following the implantation of tumour fragments from NOD-SCID in nude mice (*F1*), tumours initially appeared but a subsequent regression was observed in 3 out 5 mice (Fig. 1b). On the contrary, both NOD-SCID and SCID mice seemed to represent a suitable model for the engraftment of colon cancer PDXs, as we obtained a tumour take-rate of 60% in both mice strains, which is comparable to that reported in other studies for the same tumour histotype [17]. All these data confirm that nude mice are not profitable models to develop colon cancer PDXs. In contrast, a general consensus on the role of most immunosuppressed mice such as NSG does not exist. In fact, while a very high 83% rate was reported by Toop et al. [6] in NSG ovarian PDXs, when SCID versus NSG mice were compared, as in the case of breast cancer, a similar tumour take-rate was reported [19, 20]. In our opinion, the choice of the most appropriate animal model still remains unresolved and should be carefully investigated, considering the high costs of NSG involved.

Concerning the tumour engraftment sites, the most common site is the subcutaneous (s.c.) injection of the tumour administered through a small incision made on the dorsal region of the mice. A modification of this classical s.c. procedure has recently been proposed, consisting in the positioning of specimens in a dorsal intramuscular pocket of the mice. The authors reported that in esophageal PDX models this technique produced an improved tumour take-rate (72%) compared to the 17% observed with the standard subcutaneous method [21], attributing the higher success rate of intramuscular implants to the greater blood supply in the transplant bed. This seems to be a very convincing hypothesis but given the small number of samples evaluated, the effective superiority of this novel implantation technique should be validated in further larger studies and in different tumour histotypes.

## The predictive value of PDXs for clinical outcome

Even though many new antineoplastic compounds have shown favorable tumour responses in preclinical studies, more than 95% of novel therapeutics have failed to confirm efficacy in clinical trials. Many factors are responsible for this high failure rate, including the lack of predictive preclinical cancer models [2]. Thus, given the superiority of PDXs to classical xenografts derived from the cell lines, efforts have been made to evaluate the potential of PDXs in predicting patient response to therapy (Table 1). In a panel of seven human breast cancer patient-derived orthotopic xenografts (PDOXs), Marangoni’s group [8] investigated the response to chemotherapeutics used for the clinical management of this neoplasia, such as Docetaxel and 5-Fluorouracil (5-FU), given also in combination therapy with the monoclonal antibody Trastuzumab. The overall concordance between patients and PDOXs was 5/7 as they showed that among clinical cases, two clinical responses were observed and were concordant with xenograft sensitivity. Out of five clinical relapses or progression, three were concordant with lack of response observed in PDOXs. The authors concluded that, despite the low number of pairing cases, PDOXs could represent a promising tool for testing new agents and new anticancer protocols. Finally, a similarity between the response to the same treatment of breast cancer patients and that observed in PDOXs, was reported by Zhang et al. [19].

**Table 1:** The predictive value of PDXs for clinical outcome

Tumour histotype	Authors	Model^a^	Treatment/ molecular alterations	Corrispondence with patients^b^
Breast cancer	Marangoni et al. (ref. 8)	PDOXs (7)	Docetaxel, 5-Fluorouracil, Trastuzumab	5/7
	Zhang et al. (ref. 19)	PDOXs (10)	Docetaxel, Doxorubicin, Trastuzumab + Lapatinib	10/10
Colorectal cancer	Bertotti et al. (ref. 17)	PDXs (85)	Cetuximab, Panitumumab	85/85
Ovarian cancer	Ricci et al. (ref. 9)	PDXs (11)	Cisplatin	9/11
	Topp et al. (ref. 6)	PDXs (10)	Cisplatin	10/10
Small cell lung cancer	Anderson et al. (ref. 23)	PDXs (8)	Cisplatin, Etoposide	7/8
Colorectal cancer	Nunes et al. (ref. 7)	PDXs (52)	WT KRAS (8/52)	8/8 responded to Cetuximab
	Bertotti et al. (ref. 17)	PDXs (85)	KRAS mutated (18/85)	18/18 not responded to Cetuximab
Non-small cell lung cancer	Zhang et al. (ref.12)	PDXs (10)	EGFR mutated (1/10)	1/10 responded to Gefitinib

^a^In parentheses is reported the number of PDXs or PDOXs evaluated

^**b**^Mice were treated with the same protocol used for the patients and the response was compared

The study by Bertotti et al. [17] performed on a very large cohort of PDXs obtained from metastatic colorectal cancer, strongly supports the role of this model in mimicking the response of this disease in humans. In fact, they showed that, when unselected PDXs were treated with Cetuximab, the percentage rate regarding response (about 11%), disease stabilization (30%) and progression (59%) was in line with the data reported in the retrospective analysis of the unselected patients treated with Cetuximab or Panitumumab.

The need for better, more clinically predictive models of epithelial ovarian cancer which account for 90% of ovarian cancer, led Giavazzi’s group [9] to develop a panel of 34 PDOXs that recapitulated molecular and biological characteristics of this lethal malignancy. The established xenografts were histologically similar to the patient tumours and resembled the five main subtypes of epithelial ovarian cancer such as high-grade serous carcinoma, endometrioid carcinoma, clear cell carcinoma, mucinous carcinoma and low-grade serous carcinoma. Moreover, when engrafted intraperitoneally, xenografts reproduced the ability of human ovarian cancer to disseminate. They reported that in most PDOXs, the response to the treatment with Cisplatin matched with the corresponding patients. The paper by Topp et al. [6] confirms that ovarian cancer PDXs not only retain the phenotypic and molecular characteristics of original tumours but also resemble the clinical response to Cisplatin. In fact, the sensitivity or resistance observed in patients was consistent with the experimental data in corresponding PDXs. Interestingly, when mice bearing recurrent tumours were re-treated with a 2nd and a 3rd-line treatment, an increased resistance to Cisplatin was observed as in the clinical setting following subsequent cycle of therapy, thus confirming that this model reflects the clinical situation and could also be a very useful tool in studying the mechanisms of tumour resistance. Finally, a good correlation between the response rate to chemotherapy with Cisplatin and Etoposide combination, the approved therapeutic regimen for Small Cell Lung Cancer (SCLC), between SCLC patients and PDXs to chemotherapy, has also been reported. In particular, the authors reported only one exception to this concordance, as one PDX was resistant to the treatment, while the patient from which this PDX was derived, elicited a good response. They hypothesized that these differences could be due to establishing a more aggressive clone in mice or that additional mutations may have occured during the growth of the PDXs [22].

In the search for effective therapies for unresectable gastric cancer, experiments performed on PDXs deriving from 8 patients demonstrated that Regorafenib, a multikinase inhibitor with activity against a range of protein kinases involved in oncogenesis (KIT, RET, and RAF), angiogenesis (VEGFR1–3 and TIE2), and maintenance of the tumour microenvironment (PDGFR and FGFR), was very active in reducing the growth of tumours in mice. Looking at the mechanisms involved in the antitumoral activity of these compounds, the authors reported a reduction in angiogenesis and proliferation associated with apoptosis induction [23]. Interestingly, these results are consistent with a recent phase II trial in patients with refractory advanced gastric cancer (INTEGRATE) [24], as a significantly longer progression-free survival was observed in the Regorafenib group versus placebo.

Notably, PDXs could also be useful models for identifying predictive biomarkers of response to targeted therapies. For example, recently published studies on colon cancer PDXs confirm clinical data regarding the key role of WT *KRAS* genotype for the clinical efficacy of anti-EGFR therapy. In fact, while WT *KRAS* PDXs were sensitive to Cetuximab treatment [7], PDXs bearing *KRAS* mutations were unresponsive to the treatment [17] and these observations were concordant with the retrospective analysis in the matched patients.

The utility of PDXs in supporting a precise selection of patients for EGFR-targeted therapies such as Gefitinib has also been demonstrated [12]. These authors selected 10 PDXs from Non-Small Cell Lung Cancer (NSCLC) patient specimens and treated mice with the EGFR tyrosine kinase Gefitinib, which is known to be active in patients with NSCLC. They observed that the only one PDX model with *EGFR* Exon19 Del. activating mutation responded completely to Gefitinib, while PDXs with *KRAS* mutations or *EGFR* wild-types were insensitive to Gefitinib treatment. Even though the authors were not able to report response to Gefitinib in matched patients, these results are consistent with that reported in clinical trials [25], thus validating once again that PDX models represent powerful tools in predicting the response to anticancer therapy.

## PDXs offer a route toward personalized treatment

Evidence showing that PDXs have a high predictive power for the efficacy of standard and novel anticancer therapeutics has encouraged the idea to employ this platform in the so-called co-clinical trials, where *in vivo* preclinical studies and clinical trials could be performed in parallel, with the aim to specifically target the unique cancer of a patient or subgroup of patients. In particular, the concept of co-clinical trials refers to studies capable of defining a patient selection strategy based on molecular abnormalities or identifying mechanisms of resistance to antitumoral therapies, for the development of precision medicine aimed at personalizing anticancer treatment. In this context, PDXs could be also used as an ‘avatar’ model, in which PDXs obtained from a patient enrolled in a clinical trial could be treated with the same therapy administered to the patient, thus permitting to identify biomarkers of sensitivity or resistance to treatments.

Most intriguingly, since a major cause of failure of treatments is the acquired resistance of tumours [26], the use of novel drugs or combination could permit to select effective therapeutic strategies for second-line treatment. On this basis, Misale et al. [27] started patients with colorectal cancer on treatment with anti-EGFR inhibitors as single agents after initial response elicited relapse of the disease, due to emerging resistance. Thus, by using colon cancer PDX as a model, they investigated how the acquisition of resistance to EGFR-targeted therapies could be restrained. The *in vitro* genetic screen and functional studies on CRC showed that dual blockade of EGFR and MEK prevents acquired resistance and as a result performed experiments *in vivo* on PDXs derived from a CRC patient carrying a quadruple wild-type gene profile (KRAS, NRAS, BRAF and PIK3CA) which recapitulate the molecular profile of patients sensitive to anti-EGFR antibodies. Treatment of PDXs with the anti MEK Pimasertib alone only slightly reduced tumour growth, while Cetuximab treatment was very effective in reducing tumours to more than 70%. The subsequent regrowth of these tumours, showed resistance to drug re-challenge thus resembling the clinical findings. In contrast, the Cetuximab/Pimasertib combination elicited a complete response as tumours remained undetectable for more than 6 months. These results led authors to suggest that the use of ‘ab initio’ combination treatment could inhibit the development of resistant tumours showing molecular heterogeneity as well as highlight the usefulness of PDXs in establishing a very effective antitumoral strategy.

To search for a second-line personalized treatment in melanoma patients acquiring resistance to BRAF inhibitors, Krepler et al. [28] established 12 PDX models from–progressed patients of which 3 with NRAS mutations, MAP2K1 (MEK1) mutations in 2, BRAF amplification in 4, and aberrant PTEN in 7. Moreover, amplification of MET was observed in 3 PDXs, while re-activation of phospho-MAPK predominated at the protein level, with parallel activation of PI3K in a subset of PDXs. Treating mice with a combination of compounds acting on the different resistance mechanisms produced a marked antitumor efficacy. In particular, the triple combination treatment with Capmatinib/Encorafenib/Binimetinib, targeting MET, MAPK and PI3K pathways resulted to be more effective. This strategy which uses a different cohort of mice deriving from the same patients and bearing the same targetable mechanism of resistance, makes it possible to identify an accurate personalized therapy regimen, thus avoiding treatment failures.

A paradigmatic example of the use of PDX as an “avatar” for personalized cancer treatment is reported by Bousquet et al. [29]. In particular, a woman with a localized left breast ductal invasive triple-negative breast carcinoma treated with chemotherapy and radiation, in accordance with French national guidelines, elicited a disease relapse. PDXs obtained from metastatic nodules were analyzed by transcriptome analysis and treated with different drug combinations, resulting Paclitaxel plus Cetuximab as the most efficient therapy. Following these results in PDXs, the patient was treated with Paclitaxel plus Cetuximab and after 3 months of this second-line treatment, the metabolic response was almost complete. Moreover, the time to progression was longer than previous lines of treatment.

These examples demonstrate that PDX models integrated with targeted sequencing and phosphoproteomic platforms, provide the preclinical basis for identifying more effective therapies both in the first and second-line targeted inhibitor strategies, also for the repositioning and/or repurposing of previously approved drugs.

## Limits and challenges of PDX models

Although PDXs possess notable advantages compared to classical xenografts as described above, they do have some limitations that cannot be omitted. Importantly, overcoming these issues could increase this model’s potential for improving therapeutic application of antineoplastic compounds.

One of the major limitations of PDXs is that tumours fail to progress or to metastasize and therefore do not retain all patterns of the disease course observed in patients. One strategy to overcome this disadvantage is represented by the engraftment of tumour specimens in the orthotopic sites of origin. To this purpose, several reports have shown that patient-derived orthotopic xenografts (PDOXs) could represent a powerful tool for addressing this key point in the science of preclinical modelling. Particularly, DeRose et al. [14] reported that the establishment of breast tumours into the mammary glands of mice maintain clinical features of original tumours as the majority of mice developed metastases corresponding to patient metastatic sites, such as lymph nodes, lungs, bone and peritoneum. Moreover, Walters et al. [30] showed that PDOXs of pancreatic carcinoma developed peritoneal and liver metastasis, thus recapitulating the clinical aspects of human disease. The relevance of using PDOX in evaluating an anti- metastatic therapy is highlighted by Hiroshima et al. [31] in a model of HER2-positive cervical cancer showing peritoneal dissemination, liver, lung and lymph node metastasis. In particular, these authors observed a different sensitivity to chemotherapy between primary tumor and metastasis. In fact, while the subcutaneous model and the primary tumor of PDOX from the same tumor were not sensitive to Entinostat, a benzamide histone deactylase inhibitor, Entinostat-treatment reduced significantly the size of metastasis in comparison to mice treated with the vehicle.

At the same time the s.c. implantation does not represent the site of origin, thus limiting studies on evaluating the role of the tumour microenvironment as it is well known that the cells of the tumour vasculature, fibroblasts and inflammatory cells, interacting with tumour cells, are important in tumour biology and that the microenvironment regulates cancer-drug sensitivity [32]. Thus, the orthotopic implanting fragments directly into the organ of origin (PDOXs), requiring very skilled personnel, better recapitulates the complexity of human malignancy [33] and should be extensively adopted. In this regard, advanced real-time imaging systems permit the quantitative assessment of primary tumour growth and metastatic progression, relying on the use of imaging modalities. In relation to this, Fluorodeoxyglucose–positron emission tomography (FDG-PET) has been proposed and the feasibility of this technique has been confirmed by magnetic resonance [34, 35]. Furthermore, based on the observation that more than 90% of pancreatic cancers are EGFR-positive, a novel imaging approach which combines micro PET and F(ab’)2 fragments of the fully-human anti-EGFR monoclonal antibody, panitumumab has been evaluated [36]. The authors demonstrated that 64Cu-panitumumab F(ab’)2 fragments bound with high affinity to EGFR on PDOXs pancreatic cancer, allowed to visualize the tumour by microPET/CT. They suggest that this method could also be useful for tumour imaging in patients and for radioimmunotherapy. Finally, the use of fluorescently-labelled chimeric anti-CEA antibody permitted whole body imaging of colon cancer PDOXs. To this purpose, tumour mice bearing were injected with the anti-CEA antibody labelled with the AlexaFluor 488 and then seen by a small animal imaging system that was able to detect the primary tumour and the residual mass after the surgical resection [37].

A further limitation of the model is that mice used for engraftment have a severely compromised immune system which poses a disadvantage for the full exploitation of PDX technology, since the contribution of immune-cell function hinders the proper evaluation of tumour growth and patient response in these models. This is particularly true in certain tumours such as melanoma in which progresses in treating this neoplasia have been made with targeted immunotherapy [38, 39]. The use of the so-called humanized mice with co-engrafted stromal and immune components, will allow researchers to study tumour growth and drug response in the context of human immunosystem which could represent the most advanced preclinical models for drug efficacy studies with some aspects needing improvement [40].

**Fig. 2:**
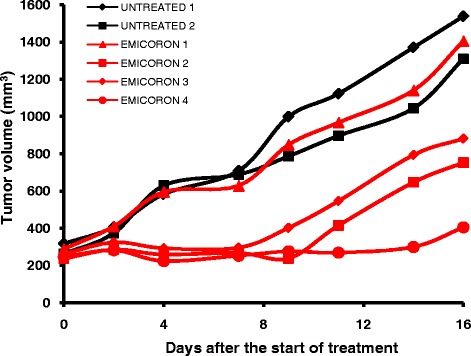
NOD-SCID mice at passage *F2* were treated with the G-quadruplex EMICORON *per os* at 15 mg/kg/day for 15 consecutive days, starting when a tumor volume of 300 mm^3^ was evident in mice. Each curve represent untreated (black) or EMICORON-treated mice (red)

The intratumour heterogeneity could have an impact on the interpretation of data stemming from PDXs as they are usually developed from small fragments obtained by larger biopsies. At the same time, the passages in mice could increase this spatial intratumoral heterogeneity since propagation is obtained by cutting the tumour mass (300–500 mm^3^) grown in one mouse and implanting small pieces of the mass (15–20 mm^3^) in several mice for propagation. Our observations seem to confirm this hypothesis as demonstrated in PDXs originating from one colorectal cancer patient and treated with a G-quadruplex (G4) ligand, one mouse showed complete response, two mice partial response followed by a rapid disease progression and no-response was elicited in one mouse (Fig. 2). These results will permit us to investigate the changes in gene expression after treatment and to predict drivers of tumour resistance to G4-ligands, thus highlighting that PDXs represent a useful model in studying intratumor heterogeneity which is one of the main reasons for the failure of treatments and disease progression.

One critical aspect concerns the maintenance of PDXs in the genetic integrity of parental tumours and the degree of alterations occurring across the passages into new mice. To this purpose there are many examples throughout literature reporting that in the initial passages, PDXs retain the histological, immunohistochemistry, gene expression, copy numbers and chromosomal stability profile of the original patient tumour [3, 4]. Modifications of tumour characteristics occurring during passages could influence the response to chemotherapy. It was for this purpose that in a recent paper, Dodbiba et al. [41] evaluated whether chemosensitivity could be modified through the passages in mice. PDXs were treated with combination treatments including Cisplatin, Paclitaxel and 5-Fluorouracil which are drugs used for the clinical management of esophageal and gastro-esophageal junction cancer. The authors observed that the inherent resistance, gain-of-resistance or chemosensitivity remained generally constant across 3 to 11 passages in mice.

In contrast, genetic changes that occurred via each passage to a new mouse host seem to represent genomic rearrangements intrinsic to tumour progression. In particular, in breast cancer PDXs it was observed that tumours in mice showed a pronounced mutational status or aggressiveness of tumour characteristics, sometimes comparable to patient metastases compared to primary tumours [20]. These observations suggest the use of PDX models with low passage numbers (<10) to preserve the genetic integrity of the parental tumour [42].

Perhaps one major obstacle in PDX modelling for co-clinical trials and personalized medicine is the long timeframe required for engraftment. In fact, the tumour latency calculated as the time between implantation and the development of a progressively growing xenograft tumour can range from 2 to 12 months. Considering that tumours have to be passaged several times in mice to generate a sufficient number of “avatars”, this means that individual patients with a rapidly progressing disease could not benefit from PDX studies. To this purpose, performing drug testing in PDXs during the period of standard therapy could be a useful strategy in view of the second-line treatment based on PDXs results.

## Conclusions

PDX models are a relevant preclinical platform and represent a significant challenge for oncology research as they, more accurately, reflect human tumour biology than any other existing models. If PDXs are viewed as complementary to other experimental models, the use of PDXs should lead to a higher rate of success in identifying new and more effective therapeutic strategies and in transitioning into individualized therapy. The aforementioned limitations of this model, the high costs associated with maintaining mouse colonies and regulatory issues have hindered the full exploitation and widespread utilization of this methodology, therefore vast efforts are needed to refine the model and overcome these challenges. The collaboration between private and public institutes devoted in the fight against cancer, in developing and characterizing large collections of PDX models from different cancer types, will accelerate progress in the area of drug development. In conclusion, the long-term benefits of using more relevant preclinical models of human tumour biology merit these efforts and expenses.
